# Impact of Ivermectin Mass Drug Administration for Lymphatic Filariasis on Scabies in Eight Villages in Kongwa District, Tanzania

**DOI:** 10.4269/ajtmh.18-0018

**Published:** 2018-07-30

**Authors:** Diana Martin, Ryan Wiegand, Brook Goodhew, Patrick Lammie, Harran Mkocha, Mabula Kasubi

**Affiliations:** 1 Division of Parasitic Diseases and Malaria, Centers for Disease Control and Prevention, Atlanta, Georgia;; 2 Kongwa Trachoma Project, Kongwa, Tanzania;; 3 Muhimbili University, Dar es Salaam, Tanzania

## Abstract

Scabies was recently added to the World Health Organization list of neglected tropical diseases. The ability to treat scabies with oral ivermectin makes a mass drug administration (MDA) campaign a feasible option for scabies control. Ivermectin MDA in communities endemic for lymphatic filariasis (LF) or onchocerciasis may already be having an impact on scabies. We examined the effect of ivermectin MDA for LF on scabies prevalence over 4 years in eight Tanzanian villages. At baseline, 4.4% (95% confidence interval [CI]: 3.7–5.4) of individuals tested positive for scabies, decreasing to 0.84% (95% CI: 0.51–1.4) after one round of ivermectin MDA but increased in Year 3 (2.5% [95% CI: 1.9–3.3]) and Year 4 (2.9% [95% CI: 2.2–3.8]). Most scabies cases were seen in children younger than 15 years. The data suggest that single-dose ivermectin MDA may not be effective in attaining long-term decreases when scabies prevalence is less than 5%.

Scabies is a skin disease caused by a mite (*Sarcoptes scabiei*) that burrows under the skin and is transmitted through prolonged skin-to-skin contact.^1^ Scabies and its complications particularly affect young children, especially in tropical developing countries.^1–3^ The direct effect of scabies is debilitating itching; excessive scratching of sores can lead to complications due to bacterial infection of the skin, including impetigo, abscesses, and cellulitis. Severe complications include septicemia, renal failure, and rheumatic heart disease.^1^ Global Burden of Disease estimates for scabies are that 250 million people worldwide are affected each year.

Treatment guidelines in most countries call for treatment of uncomplicated scabies with topical creams such as permethrin, benzyl benzoate, 5–10% sulfur in paraffin, or crotamiton; treatment of the affected person’s family members is recommended.^4^ Single-dose ivermectin is also an effective treatment, although a second dose of ivermectin may be required for optimal efficacy. Ivermectin has been widely used as a preventive chemotherapeutic agent in mass drug administration (MDA) campaigns for lymphatic filariasis (LF) elimination programs throughout Africa and for onchocerciasis in the Americas.^5,6^ Some studies have shown collateral benefits of ivermectin MDA for LF on scabies^7,8^ but further studies are needed to formally demonstrate this association. We evaluated the impact of annual ivermectin MDA given for LF, following World Health Organization (WHO) guidelines,^9^ on the community-wide prevalence of scabies in eight treatment-naive villages in Kongwa district, Tanzania.

This study adhered to the guidelines of the Declaration of Helsinki. Ethical approval for the study was granted by the Institutional Review Board of the Institute for Medical Research Ethical Review Committee in Dar es Salaam, United Republic of Tanzania, and the Centers for Disease Control and Prevention, Atlanta, GA. Written informed consent was obtained from all individuals aged 18 years and older. Parental consent was obtained for all children aged 1–18 years and verbal assent was obtained from children aged 6–17 years. No serious adverse events were associated with MDA.

The study was conducted as a series of yearly cross-sectional studies. This study was one component of a larger health impact study for which sample size calculation was based on trachoma prevalence in 1–9 year olds.^10^ Ninety-six balozis (neighborhood units of approximately 10 houses each) were selected for the study, with a predicted 20 children of age 1–9 years per balozi and one additional individual older than 10 years per household for a total sample size of 3,920 (including 1,860 1–9 year olds). At baseline and each year of follow-up, a new census was conducted and a new random sample for enrollment in the study was selected. Baseline data collection occurred from October to December 2012 with follow-up data collection in October–December each year until 2015. Mass drug administration was given approximately 1–2 weeks after field data collection in each year to the entire population. Mass drug administration coverage was > 85% each year. Data were entered using the LINKS system^11^ and downloaded to a Microsoft Excel spreadsheet (Microsoft Corp., Redmond, WA). Data were cleaned in Excel and analyzed in R version 3.3.2 (R Core Team, Vienna, Austria) using the survey^12^ and ggplot2^13^ packages. Confidence intervals were calculated using the incomplete beta function.^14^

Demographic data for individuals with age and scabies data are presented in Table 1. More females than males were enrolled each year (55.4% female at baseline, 54.5% in Year 2, 55.0% in Year 3, and 54.0% in Year 4). The number of enrolled individuals is shown in Table 1 stratified into three age groups; enrollment of persons ≥ 10 years old was poor each year.

**Table 1:** Demographic characteristics of individuals enrolled in scabies evaluation at each year

	Baseline (2012)	Year 2 (2013)	Year 3 (2014)	Year 4 (2015)
Gender	*N* (%)	*N* (%)	*N* (%)	*N* (%)
Male	1,012 (44.6)	919 (45.5)	923 (45.0)	948 (46.0)
Female	1,257 (55.4)	1,102 (54.5)	1,129 (55.0)	1,111 (54.0)
Age range (years)	*N* (%)	*N* (%)	*N* (%)	*N* (%)
1–9	1,633 (72.0)	1,501 (74.3)	1,557 (75.9)	1,481 (71.9)
10–14	137 (6.0)	140 (6.9)	121 (5.9)	166 (8.1)
≥ 15	499 (22.0)	380 (18.8)	374 (18.2)	412 (20.0)
Total participants	2,269	2,021	2,052	2,059

Gender and age are shown by year of the study. Numbers in parentheses represent the percent of individuals in each group out of the total participants in each year.

Community health workers were trained in clinical diagnosis of scabies and were given a flip chart with images of scabies on dark skin and images of other skin conditions that were not to be recorded as scabies. The presence of itching and of infected scabies (i.e., the presence of ulceration around the scabies lesion) was also recorded. Only exposed skin and extremities (arms, hands, legs, and feet) were examined. Scabies rates of all ages dropped from 4.4% at baseline to 0.84% in Year 2 and then increased to 2.5% in Year 3 and 2.9% in Year 4 (Table 2). Of 229 recorded cases of scabies over 4 years, 167 (72.9%) were recorded as infected scabies. The increase in scabies rates at the Year 3 follow-up was primarily related to a single village (Village 4, Figure 1). Teams revisited this village to determine a source of possible reintroduction, such as a number of new families moving in, or a case of crusted scabies that could reintroduce scabies to the community but were not able to determine a specific cause for this increase in Year 3.

**Table 2:** Scabies rates by age range

	Baseline (2012)	Year 2 (2013)	Year 3 (2014)	Year 4 (2015)
Age range	No. positive (% [95% CI])	% (95% CI)	% (95% CI)	% (95% CI)
1–9	90 (5.5 [4.5–6.8])	14 (0.93 [0.53–1.6])	45 (2.9 [2.1–3.9])	55 (3.7 [2.8–4.8])
10–14	8 (5.8 [3.0–11.1])	2 (1.4 [0.25–5.6])	3 (2.5 [0.6–7.6])	4 (2.4 [0.77–6.4])
≥ 15	3 (0.6 [0.2–1.9])	1 (0.26 [0.01–1.7])	3 (0.8 [0.21–2.5])	1 (0.24 [0.01–1.6])
All ages	101 (4.4 [3.7–5.4])	17 (0.84 [0.51–1.4])	51 (2.5 [1.9–3.3])	60 (2.9 [2.2–3.8])

Data are shown as the number of positive scabies cases. Parentheses show the percentage with a positive scabies result of the total number of individuals in that age range and 95% confidence intervals (CIs).

**Figure 1.**
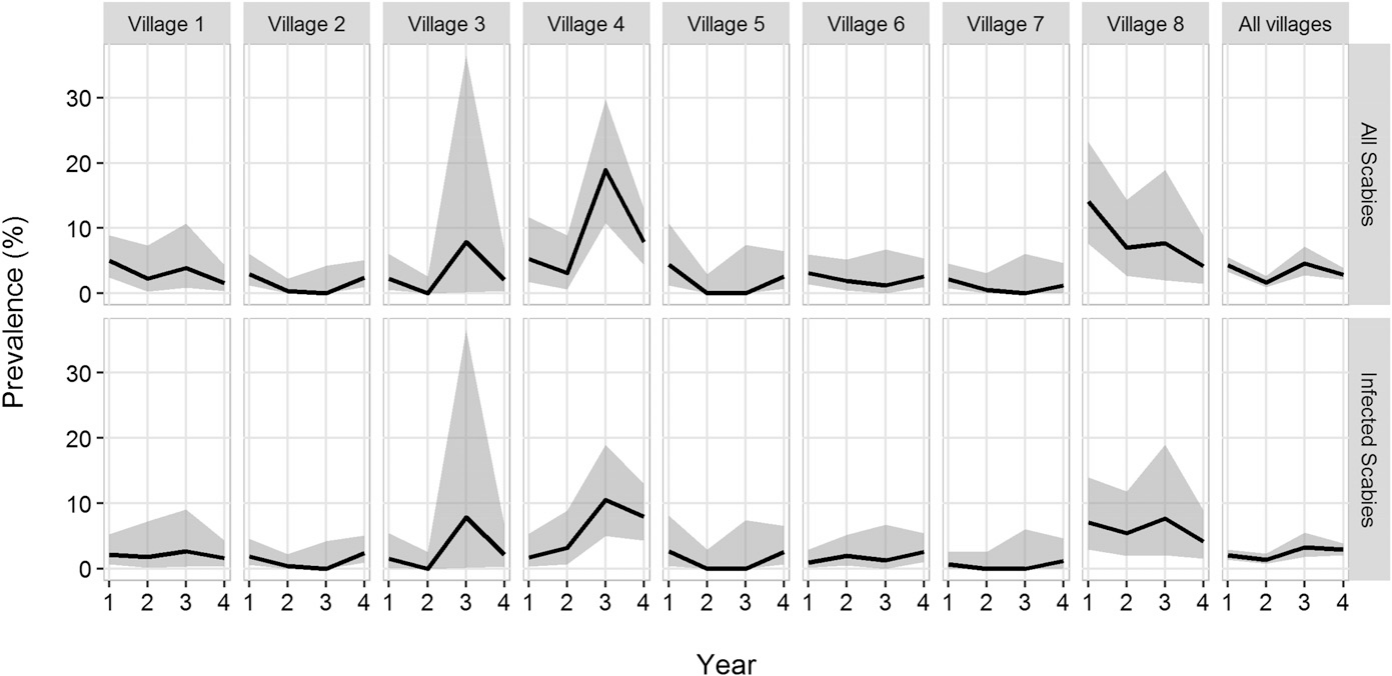
Prevalence and 95% confidence intervals (CIs) of all scabies and active scabies by year for all villages individually and overall. Prevalence trajectories are represented by the black lines and 95% CIs by gray bands.

At baseline, swabs were taken from scabies sores and bacterial analysis was carried out in the Department of Microbiology/Immunology, Muhimbili National Hospital, Dar es Salaam. Specimens were inoculated onto blood, MacConkey, and chocolate agar plates, incubated in 5% CO_2_ at 37°C, and then examined after 24 hours. Specimens with no obvious growth were incubated for an additional 24 hours and colonies rescreened after 48 hours. Suspected bacterial organisms were identified on the bases of their colonial morphology, and Gram staining and appropriate biochemical tests were conducted using standard techniques^15^ according to the guidelines of the Clinical and Laboratory Standards Institute (CLSI 2013). At baseline, 57/92 (62.0%) swabs from scabies sores were culture positive for *Staphyloccocus aureus*. None tested positive for *Streptococcus* spp., an unanticipated result but likely related to the difficulties in transporting specimens in a timely manner (2–3 times weekly) from the field site to the laboratory in Dar es Salaam. Because of the length of time it took in transporting skins swabs to the laboratory in Dar es Salaam from field sites and maintaining the proper temperature during transport, we discontinued collecting these in 2014.

Aside from the scabies swab shipments, there were several other limitations to this study. Poor recruitment of ≥ 10 year olds means samples do not represent a community sample. If adults have fewer infections than children, we may be overestimating the community-wide prevalence of scabies. Communities were selected as study sites based on the prevalence of trachoma, not scabies, which may impact the outcome. The study was not cluster-randomized, and only evaluated changes over time, as we could not ethically withhold the treatment of scabies or LF in a control population. We only examined extremities for scabies, not the full body, so some scabies may have missed if presenting only on torso or buttocks. However, all individuals were asked about itching and none responded “yes” to itching without a corresponding clinical scabies diagnosis on the extremities.

These data are consistent with those from a staged MDA rollout in an Aboriginal population of Australia, where a population prevalence of scabies dropped from 4% at baseline to 1% at 6 months and then increased to 9% at 12 months.^16^ These data were confounded by local cases of crusted scabies, which was not seen in our study communities. By contrast, mass drug trials in Solomon Islands (children < 12)^17^ and Fiji (all ages)^18^ showed a dramatic impact on scabies prevalence: ivermectin MDA decreased community-wide scabies prevalence from 25% to < 1% and 32.1% to 1.9%, respectively. The different outcomes may relate to the use of different drug treatment regimens in Solomon Islands and Fiji, where two doses of ivermectin were given 7–14 days apart. Individuals for whom ivermectin was contraindicated (primarily children < 15 kg and women who were pregnant or breastfeeders) were given permethrin cream. In our study, a high proportion of scabies cases were younger than 5 years, who were offered benzyl benzoate cream rather than ivermectin, and this treatment was not directly observed. In a similarly high-prevalence village in Papua New Guinea,^7^ following a single dose of ivermectin scabies prevalence decreased from 87.1% to less than 10% in 2 months, leveling off to 26% at 5 months after treatment. In Zanzibar,^8^ the number of scabies cases registered at primary health-care units decreased over 5 years following implementation of ivermectin MDA, although cases continued to be reported throughout the evaluation and no prevalence data were collected. These reports suggest that interventions may have the highest impact in high-prevalence communities and that intensive follow-up may be needed to attain sustained control of scabies. The effect of mass treatment on non-island populations also needs to be evaluated further.

Scabies was recently added to the WHO list of neglected tropical diseases (http://www.who.int/neglected_diseases/NTD_STAG_report_2017.pdf?ua=1), and there is growing interest in worldwide scabies control.^19–21^ The data here suggest that after an initial decrease in scabies rates following ivermectin MDA, community-wide scabies rates leveled off and did not continue dropping, at least in young children. There are currently little data to guide potential program targets or determine what prevalence of scabies constitutes a public health problem. The data presented here may help with this guidance in showing that single-dose ivermectin interventions may have a limited long-term impact on scabies rates in settings where the prevalence is less than 5%.
